# Thrombotic Events in a Patient With Acute Toxoplasmosis

**DOI:** 10.7759/cureus.69797

**Published:** 2024-09-20

**Authors:** Pedro Arthur Da Rocha Ribas, João Álvaro Da Rocha Ribas, Rafael Mialski Fontana

**Affiliations:** 1 Internal Medicine, Clinical Hospital Complex of the Federal University of Paraná, Curitiba, BRA; 2 Family and Community Medicine, Municipal Health Department, Manaus, BRA; 3 Infectious Disease Service, Clinical Hospital Complex of the Federal University of Paraná, Curitiba, BRA

**Keywords:** inflammation, toxoplasmosis, portal vein thrombosis, pulmonary thromboembolism, venous thromboembolism

## Abstract

The etiology of venous thromboembolism is multifactorial, with causes being classified as either provoked or unprovoked, making it difficult to attribute a single factor as the cause of the thromboembolic event in most cases. The relationship between inflammation and thrombotic phenomena is well established. Here, we describe the uncommon occurrence of thromboses in a previously healthy patient with acute toxoplasmosis. The patient initially presented with fatigue, abdominal pain, fever and dyspnea. The diagnosis was confirmed through toxoplasmosis serology in a set of admission laboratory tests, and further imaging studies revealed the presence of pulmonary embolism and portal vein thrombosis. The patient was treated with anticoagulants and sulfamethoxazole-trimethoprim, showing improvement in the following days. This case highlights the importance of considering infectious diseases, such as toxoplasmosis, in the differential diagnosis of thrombosis, even in previously healthy individuals. To our knowledge, this is the second reported case of this association in Brazil.

## Introduction

Venous thromboembolism (VTE), which includes deep vein thrombosis (DVT) and pulmonary embolism (PE), has an annual incidence of approximately 1 in 1,000 people [1]. The etiology of VTE is multifactorial, with causes being classified as either provoked (such as immobilization, surgeries, inflammation) or unprovoked, making it difficult to attribute a single factor as the cause of the thromboembolic event in most cases [2,3].

PE involves the obstruction of the pulmonary artery and/or its branches by emboli originating from thrombi in the systemic venous circulation. The main clinical manifestations include dyspnea, tachypnea, chest pain, and tachycardia [3]. Treatment of PE is done with anticoagulants and is divided into initial phase (days to weeks), primary treatment (three to six months), and secondary prevention (indication guided by the cause) [4]. Portal vein thrombosis (PVT) is characterized by primary obstruction by a thrombus located in the trunk or branches of the portal vein. It is estimated that in 75% of patients with PVT who do not have an underlying liver disease, a risk factor for venous thrombosis can be identified, which can be divided into local (such as inflammatory conditions) and systemic risk factors (such as thrombophilias) [5]. The development of PVT in non-cirrhotic patients is a rare condition. According to the American Association for the Study of Liver Diseases (AASLD), obstruction of the extrahepatic portal venous system in non-cirrhotic patients is often related to myeloproliferative neoplasms, surgeries or inflammatory conditions [6].

Toxoplasmosis, a zoonosis caused by the intracellular protozoan *Toxoplasma gondii*, is a condition primarily acquired through the ingestion of raw meats contaminated with cysts of the agent. It is estimated that 30% of the population worldwide is chronically infected with *T. gondii *[7]. Some of the classic clinical manifestations of acute toxoplasmosis include fever, chills, night sweats, loss of appetite, and lymphadenopathy [8]. The inflammatory process, whether of infectious or non-infectious origin, is related to the risk of developing thrombotic phenomena. In the presence of inflammation, there is activation of endothelial cells, leukocytes, platelets, and the coagulation cascade, increasing the risk of thrombus formation [9]. The gold standard treatment for toxoplasmosis is the combination of sulfadiazine with pyrimethamine, though other treatments may also be used, such as sulfamethoxazole-trimethoprim [10].

## Case presentation

A 64-year-old previously healthy White male, with no history of smoking or alcohol use, and not on any continuous medications, was admitted to a tertiary care hospital in October 2022 for elective umbilical hernia repair without complications and was discharged the same day. Approximately 15 days after surgery, he developed fatigue, mild abdominal pain, loss of appetite, daily afternoon fever of 38°C, night sweats, and dyspnea with moderate exertion, but denied cough or other complaints. Three days after the onset of symptoms, the patient experienced a syncopal episode while waiting in an airport lounge for a flight from Curitiba to São Paulo (a flight lasting approximately one hour), but did not seek medical attention during the time he was away from his home city. After seven days of symptom onset, now back in Curitiba, he sought emergency care due to the persistence of his complaints, which now included worsening fever patterns that had become constant and more intense, reaching temperatures of up to 39.5°C.

During the initial medical evaluation, a physical examination revealed no inflammatory signs at the surgical incision or any other abnormalities. Vital signs were stable, with a peripheral oxygen saturation of 97% at rest. A chest X-ray showed no changes, and a rapid SARS-CoV-2 test (COVID-19) was negative (the patient had already received three doses of the COVID-19 vaccine: two doses of AstraZeneca and one dose of Pfizer, all administered over two years prior). He was discharged from the emergency department with instructions and symptomatic medications.

**Figure 1:**
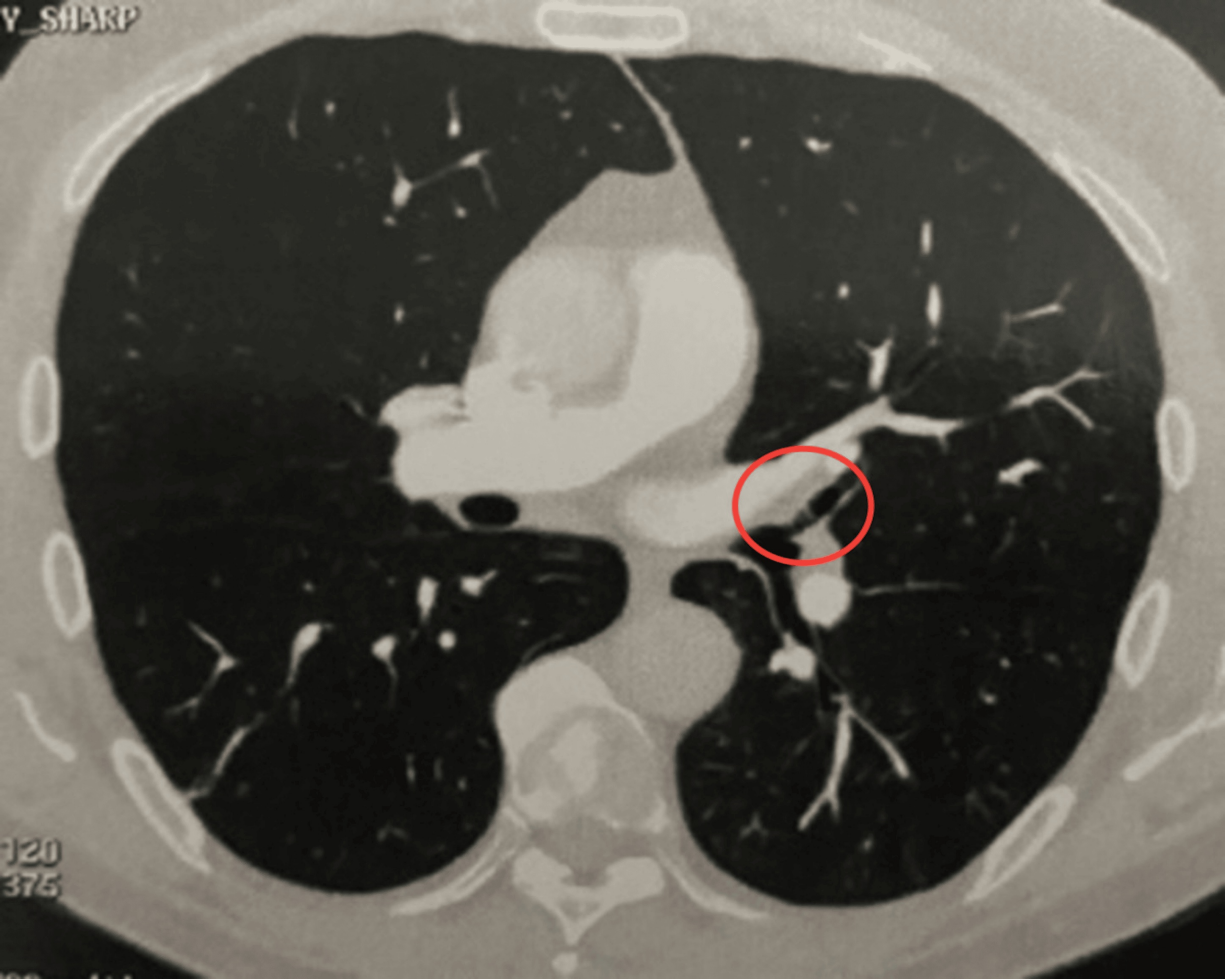
Filling defect in the lower segment of the left pulmonary artery

On the 10th day after the onset of symptoms, due to the persistence of symptoms, he sought medical attention again and was admitted for diagnostic investigation. During hospitalization, no abnormalities were observed on physical examination, including no signs of deep vein thrombosis. Admission laboratory tests showed elevated liver transaminases and positive IgM for toxoplasmosis (Table 1). A thoracic CT angiography, carried out due to a high pre-test probability of PE in a patient with dyspnea (Wells score, 4.5 points), revealed a PE in the lower segment of the left pulmonary artery (Figure 1). Anticoagulation with enoxaparin was initiated. Upon questioning, the patient reported having consumed raw kibbeh at a restaurant in Curitiba 10 days before the onset of symptoms.

**Table 1:** Admission laboratory tests

Parameters	Value	Reference Range
Hemoglobin	14.2 g/dl	12-17.5 g/dl
Leukocytes	9,120/mm^3^	3,500-10,500/mm^3^
Platelets	299,000/mm^3^	150,000-450,000/mm^3^
C-reactive protein (CRP)	54.4 mg/l	<10 mg/l
Urea	28.0 mg/dl	19.0-43.0 mg/dl
Creatinine	1.2 mg/dl	0.8-1.5 mg/dl
Total bilirubin	1.04 mg/dl	0.2-1.48 mg/dl
Indirect bilirubin	0.38 mg/dl	≤1.3 mg/dl
Direct bilirubin	0.66 mg/dl	≤0.5 mg/dl
Alkaline phosphatase	152 U/l	38-126 U/l
Gamma-glutamyl transferase (GGT)	215 U/l	15-73 U/l
Aspartate aminotransferase (AST)	77 U/l	17-59 U/l
Alanine aminotransferase (ALT)	79 U/l	11-66 U/l
Anti-HIV	Non-reactive	Non-reactive
Dengue IgM	Non-reactive	Non-reactive
Cytomegalovirus IgM	0.2	Reagent: ≥1
Epstein-Barr virus IgM	15	Reagent: ≥40
Toxoplasmosis IgM	33	Reagent: ≥1

**Figure 2:**
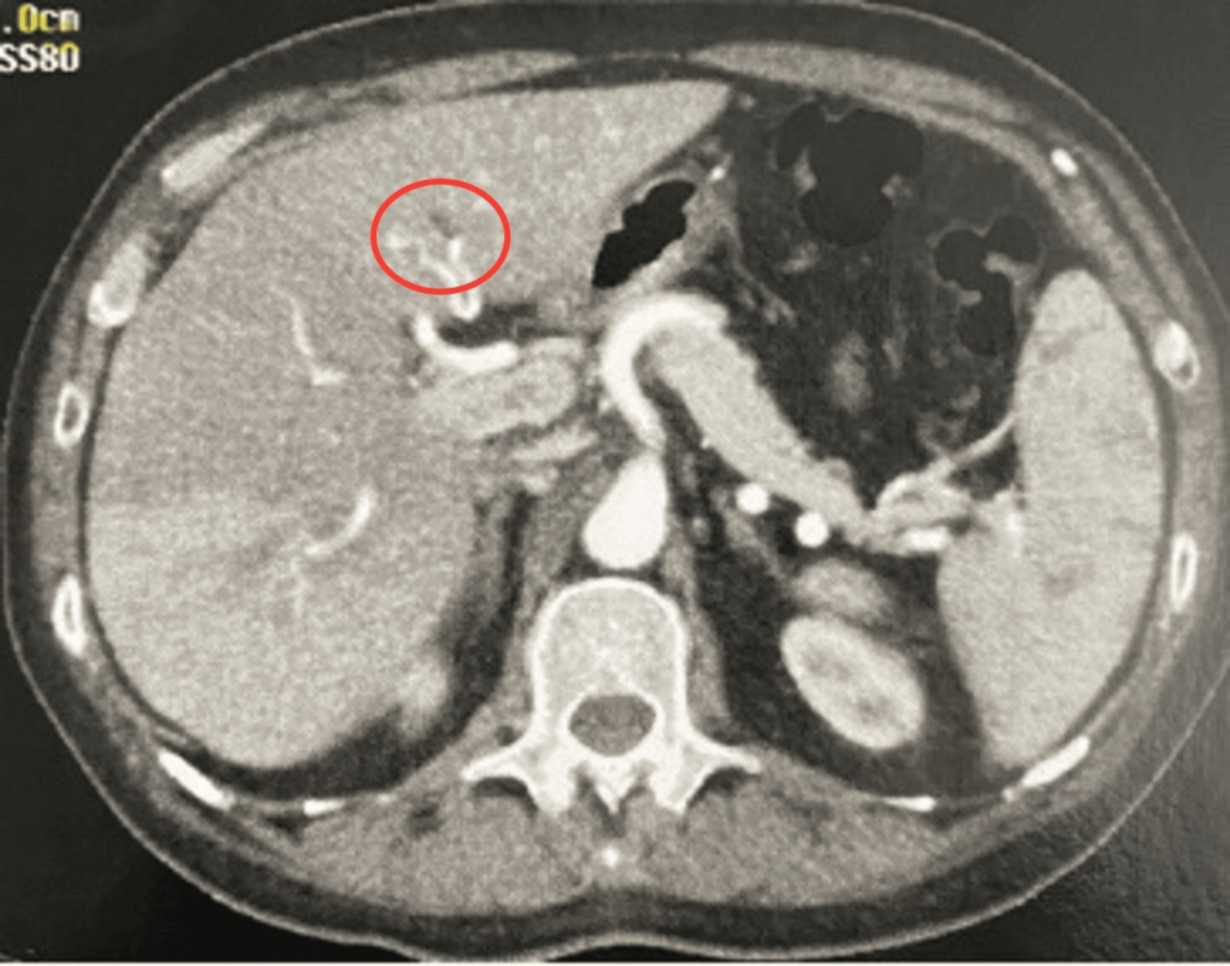
Filling defect with hypoattenuating material within the portal segment of the left hepatic lobe (segment IV)

To complement the diagnostic investigation, the following imaging tests were also performed, showing no abnormalities: venous Doppler ultrasound of the lower limbs, transthoracic echocardiography, upper gastrointestinal endoscopy, colonoscopy, and magnetic resonance cholangiopancreatography (Table 2). During hospitalization, due to the progressive rise in liver transaminases (Table 3), an abdominal CT angiography was performed, revealing thrombosis in the portal system (Figure 2).

**Table 2:** Imaging tests and results

Tests	Results
Thoracic CT angiography	Filling defect by contrast agent in subsegmental branches of the right lower lobar pulmonary artery
Venous Doppler ultrasound of lower limbs	Negative for deep vein thrombosis (DVT)
Transthoracic echocardiography	Left ventricular ejection fraction of 63%, normal-sized cardiac chambers, preserved systolic and diastolic functions of the left ventricle, preserved systolic function of the right ventricle, no signs of pulmonary hypertension, normal valves
Upper gastrointestinal endoscopy	Within normal parameters
Colonoscopy	Within normal parameters
Magnetic resonance cholangiopancreatography	No dilation of intra- or extrahepatic bile ducts. Normal pancreas dimensions, regular contours, and no dilation of the Wirsung duct
Abdominal CT angiography	Filling defect with hypoattenuating material within the portal segment of the left hepatic lobe (segment IV)

**Table 3:** Laboratory control tests

Parameters	Value	Reference Range
Hemoglobin	13.1 g/dl	12-17.5 g/dl
Leukocytes	8,750/mm^3^	3,500-10,500/mm^3^
Platelets	329,000/mm^3^	150,000-450,000/mm^3^
C-reactive protein (CRP)	42.0 mg/l	<10 mg/l
Urea	25.0 mg/dl	19.0-43.0 mg/dl
Creatinine	1.1 mg/dl	0.8-1.5 mg/dl
Total bilirubin	0.89 mg/dl	0.2-1.48 mg/dl
Indirect bilirubin	0.24 mg/dl	≤1.3 mg/dl
Direct bilirubin	0.65 mg/dl	≤0.5 mg/dl
Alkaline Phosphatase	157 U/l	38-126 U/l
Gamma-glutamyl transferase (GGT)	265 U/l	15-73 U/l
Aspartate aminotransferase (AST)	245 U/l	17-59 U/l
Alanine aminotransferase (ALT)	174 U/l	11-66 U/l
Prostate-specific antigen (PSA)	0.99 ng/ml	≤4.0 ng/ml

**Figure 3:**
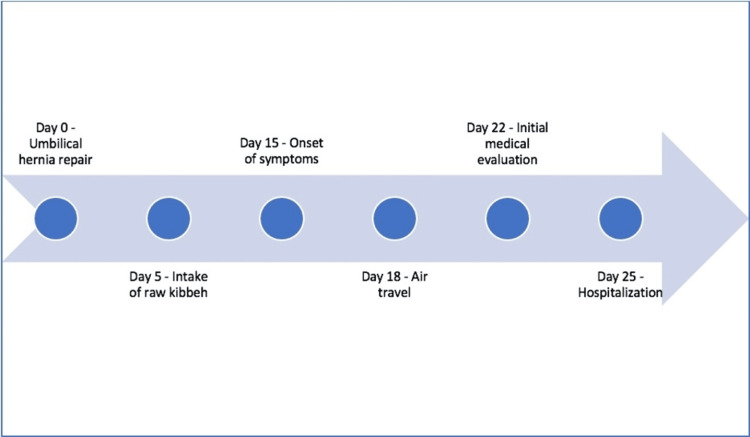
Case report timeline

After six days of hospitalization with clinical and laboratory improvement, he was discharged with rivaroxaban for six months and sulfamethoxazole-trimethoprim for 21 days, continuing follow-up on an outpatient basis with an infectious disease specialist and hematologist (Figure 3). During outpatient consultations, four weeks after hospital discharge, tests for thrombophilia (Table 4) were conducted, which returned negative results, and new laboratory control tests were carried out (Table 5).

**Table 4:** Thrombophilia investigation

Tests	Results
Factor V Leiden mutation	No Factor V Leiden mutation
JAK2 V617F mutation	No V617F mutation
Lupus anticoagulant	Negative for lupus anticoagulant
Anticardiolipin IgM	Non-reactive
Anticardiolipin IgG	Non-reactive

**Table 5:** Laboratory control tests four weeks after hospital discharge

Parameters	Value	Reference Range
Hemoglobin	15.3 g/dl	12-17.5 g/dl
Leukocytes	5,750/mm^3^	3,500-10,500/mm^3^
Platelets	174,000/mm^3^	150,000-450,000/mm^3^
Urea	25.0 mg/dl	19.0-43.0 mg/dl
Creatinine	0.93 mg/dl	0.8-1.5 mg/dl
Total bilirubin	0.85 mg/dl	0.2-1.48 mg/dl
Indirect bilirubin	0.56 mg/dl	≤1.3 mg/dl
Direct bilirubin	0.29 mg/dl	≤0.5 mg/dl
Alkaline phosphatase	80 U/l	38-126 U/l
Gamma-glutamyl transferase (GGT)	42 U/l	15-73 U/l
Aspartate aminotransferase (AST)	28 U/l	17-59 U/l
Alanine aminotransferase (ALT)	25 U/l	11-66 U/l
Toxoplasmosis IgM	>160	Reagent: ≥1
Toxoplasmosis IgG	>650	Reagent: ≥3

## Discussion

A systematic review showed that infections, in general, increase the risk of developing VTE [11]. The increase in inflammatory markers such as C-reactive protein (CRP) has also been associated with a higher risk of developing this condition [12]. Another study showed the presence of thrombotic events in two patients with acute toxoplasmosis who had a heterozygous Factor V Leiden mutation, which was apparently the first reported case of such association [9]. Similarly, the patient in this case report also had positive serology for acute toxoplasmosis, but no hereditary factors for thrombophilia.

Surgeries are also well-established risk factors for PE, with a higher or lower risk depending on the type of surgery the patient underwent [2]. Among the surgeries with the highest risk of developing PE postoperatively are hip and knee arthroplasties [13]. The highest risk of developing PE after abdominal hernia repair surgeries occurs mainly in male patients over 60 years old, obese, and undergoing longer and more complex surgeries requiring hospitalization for more than one day [14]. Among the main factors mentioned, our patient had only two: male gender and age over 60 years.

According to the 2020 guideline from the American Gastroenterological Association (AGA), thrombophilia is the most common cause of PVT in non-cirrhotic patients [15]. PVT is a rare complication in the postoperative period of abdominal surgery. A study by Kim et al. analyzed patients who underwent colorectal cancer resection and found a PVT incidence of just 0.24% among 4,232 patients [16]. Although PVT can be developed in the postoperative context of abdominal surgeries, its incidence in the postoperative period is very uncommon, and thrombophilia, the most common cause of PVT in non-cirrhotic patients, was excluded in our patient.

Among the possible causes for the thrombotic events presented by the patient, one could be related to neoplasms. The mechanism of thrombosis in cancer patients can be the morbidity itself, as well as its treatment and complications [17]. However, in the present case report, neoplasms were excluded through screening tests.

Another possible cause of thrombosis could be related to COVID-19 vaccines. The AstraZeneca vaccine has been associated with a rare complication that triggers thromboembolic events in patients who received this vaccine, known as vaccine-induced immune thrombotic thrombocytopenia (VITT), which typically develops two weeks after vaccination. Although well-established, this complication is uncommon [18]. Furthermore, the patient in question had been vaccinated over two years ago and had not developed thrombocytopenia or other complications after vaccination.

Regarding thromboembolic phenomena triggered by travel, studies have shown a directly proportional relationship between travel hours and the risk of thrombosis. The WHO Research Into Global Hazards of Travel (WRIGHT) project showed that the risk of PE after flights lasting four hours or more was 1 in 6,000 healthy individuals [19]. In this case report, our patient undertook a journey of approximately one hour and was already experiencing symptoms before the trip.

Regarding hereditary factors, studies have shown that approximately 20% of patients diagnosed with a first episode of VTE had the Factor V Leiden mutation present [20]. The presence of the Factor V Leiden mutation is the most common genetic cause of thrombophilia in European populations, but it is less common in non-European populations [19]. However, hereditary causes of thrombophilia were excluded in our patient.

## Conclusions

Inflammatory processes are associated with an increased incidence of thromboembolic phenomena. We report an unusual clinical case of a patient with acute *Toxoplasma gondii *infection who developed PE and portal vein thrombosis in the context of late postoperative umbilical hernia repair. Given the multifactorial nature of thrombosis and the exclusion of other potential causes, it is suggested that a synergistic effect between toxoplasmosis and the postoperative period likely contributed to the thrombotic events in this case. Thromboses are rare but potentially severe complications of infectious processes. Therefore, this case emphasizes the need for a detailed clinical evaluation for atypical presentations of common diseases, such as thrombosis in patients presenting with acute toxoplasmosis, specially if there are any signs or symptoms suggestive of VTE.
